# Impact of the iWHELD digital person‐centered care program on quality of life, agitation and psychotropic medications in people with dementia living in nursing homes during the COVID‐19 pandemic: A randomized controlled trial

**DOI:** 10.1002/alz.13582

**Published:** 2023-12-20

**Authors:** Joanne McDermid, William Henley, Anne Corbett, Gareth Williams, Jane Fossey, Linda Clare, Chris Fox, Dag Aarsland, Zunera Khan, Maria Soto, Barbara Woodward‐Carlton, Esme Moniz Cook, Jeffrey Cummings, Adrienne Sweetnam, Xavier Chan, Megan Lawrence, Clive Ballard, the iWHELD study group

**Affiliations:** ^1^ University of Exeter Medical School University of Exeter Exeter UK; ^2^ Institute of Psychiatry Psychology and Neuroscience King's College London London UK; ^3^ NIHR Applied Research Collaboration South‐West Peninsula University of Exeter Exeter UK; ^4^ Research and Clinical Alzheimer's Disease Center CMRR Gérontopôle, CHU Toulouse, AGING team axe MAINTAIN CERPOP Toulouse France; ^5^ Alzheimer's Society UK London UK; ^6^ Faculty of Health Sciences University of Hull Hull UK; ^7^ Chambers‐Grundy Center for Transformative Neuroscience, Department of Brain Health, School of Integrated Health Sciences University of Nevada Las Vegas (UNLV) Las Vegas Nevada USA

**Keywords:** agitation, COVID‐19, dementia, digital, iWHELD, nursing home, person‐centered care, psychotropic, quality of life

## Abstract

**INTRODUCTION:**

iWHELD is a digital person‐centered care program for people with dementia in nursing homes adapted for remote delivery during the COVID‐19 pandemic.

**METHODS:**

A 16‐week two‐arm cluster‐randomized controlled trial in 149 UK nursing homes compared iWHELD with treatment as usual (TAU). Primary outcome was the overall quality of life with secondary outcomes of agitation and psychotropic use.

**RESULTS:**

iWHELD conferred benefit to quality of life on the primary (F = 4.3, *p* = 0.04) and secondary measures of quality of life (F = 6.45, *p* = 0.01) and reduced psychotropic medication use (*χ* ^2^ = 4.08, *p* = 0.04) with no worsening of agitation. Benefit was seen in participants who contracted COVID‐19, those with agitation at baseline, and those taking psychotropic medications.

**DISCUSSION:**

iWHELD confers benefits to quality of life and key measures of well‐being, can be delivered during the challenging conditions of a pandemic, and should be considered for use alongside any emerging pharmacological treatment for neuropsychiatric symptoms.

**Highlights:**

- iWHELD is the only remote, digital delivery nursing home training programme for dementia care
- iWHELD improved quality of life in people with dementia and reduced antipsychotic use without worsening of agitation
- Residents who contracted Covid‐19 during the study also experienced benefits from iWHELD
- iWHELD offers a valuable, pandemic‐safe tool for improving dementia care

## 1 INTRODUCTION

The Covid‐19 pandemic had a devastating impact on society. The repercussions were felt across all communities but were especially challenging for people with dementia living in nursing homes, where the impacts on health were severe.^1^ Detrimental impacts on quality of life (QoL) and neuropsychiatric symptoms over this period are also highly likely but were difficult to research because of the pandemic restrictions. Prior to the pandemic, as a result of concerted efforts in Europe and North America, there had been a substantial reduction in antipsychotic prescriptions from 40% to 50% in 2001 to under 20% by 2018.^2^, ^3^ There were, however, concerning reports of increased prescribing of antipsychotic and other sedative psychotropic medications for people with dementia during the COVID‐19 pandemic.^4^, ^5^

Agitation, especially involving physical or verbal aggression, is frequently the most challenging neuropsychiatric symptom amongst people with dementia living in nursing homes.^6^ Most studies report that 40% to 60% of nursing home residents with dementia have agitation, resulting in distress to the residents themselves and creating significant challenges for nursing home staff and clinical management.^7^ There have been few studies examining agitation in nursing homes during the pandemic. Our work indicates that although the frequency of agitation has not increased, there has been an increase in the use of pharmacological management with psychotropic medications including atypical antipsychotics in nursing home settings.^8^

The increased prescribing of antipsychotic medications for people with dementia during the pandemic is a significant concern. It is well established that atypical antipsychotics are associated with an increased risk of mortality, falls and fractures, pneumonia and stroke in people with dementia.^9^ Although there may be some adaptations of clinical practice following the Food and Drug Administration's approval of Brexpiprazole for the treatment of agitation in the US,^10^ with further approvals in Europe likely, excess mortality remains a concern^10^ and the same principles of judicious prescribing will apply that are central to the use of other atypical antipsychotics for people with dementia.

Emerging evidence related to other psychotropic medications highlights many of the same risks, particularly falls and fractures.^11^ In addition, although atypical antipsychotics have had a modest impact on reducing neuropsychiatric symptoms such as agitation, treatment has been associated with a worsening in QoL.^12^ Over the last two decades concerted efforts in the US and internationally have substantially reduced the use of antipsychotic medication and other key psychotropic agents such as hypnotics and anxiolytics.^13^ Whilst it is important to acknowledge the increased pressures of the pandemic, it is imperative to introduce measures to return the level of prescribing of antipsychotics and psychotropic medications to pre‐pandemic rates to avoid detrimental impacts on health and well‐being. In addition, research to date has centered on the impact of the pandemic on nursing homes but there has been less attention on the direct impact on individuals residing in nursing homes who have experienced COVID‐19 infection and the effectiveness of interventions to improve QoL within this group of individuals.

There is a strong evidence base to support the value of psychosocial intervention programs focusing on person‐centered care (PCC) and personalized activities in nursing home residents with dementia.^14^, ^15^ For example, the Improving Well‐being and Health for People With Dementia (WHELD) program, delivered through in‐person training, has improved QoL, reduced agitation, and decreased antipsychotic drug use across two large randomized clinical trials (RCTs) with more than 1000 nursing home patients. To address the pressing needs arising from the pandemic WHELD was adapted for remote delivery to create iWHELD, a first‐of‐its‐kind program that combines personalized care, virtual coaching, and a digital hub. iWHELD's usability and acceptability were established in a pilot RCT in 130 nursing home residents, which also reported benefits to QoL.^16^

This study delivers a definitive RCT of the iWHELD program to establish its impact on QoL, agitation, and psychotropic prescribing in nursing home residents with dementia during the COVID‐19 pandemic including an evaluation of the impact on individuals who contracted COVID‐19.

## 2 METHODS

### 2.1 Study design

This was a 16‐week two‐arm cluster‐randomized controlled trial conducted in 149 UK nursing homes during the COVID‐19 pandemic with participant recruitment from March 2021 to June 2022. The study received ethical approval from West Midlands – Coventry and Warwickshire Research Ethics Committee (Ref: 20/WM/0289) and is registered at clinicaltrials.gov (Ref: NCT04590469). Each cluster was randomized to receive either the optimized iWHELD intervention or treatment as usual (TAU) for 16 weeks.

### 2.2 Eligibility criteria

All nursing homes providing care for people with dementia across the eight regions of England were eligible unless they were under special measures for poor performance from inspection by the UK Care Quality Commission. Within each participating nursing home, all residents were considered potentially eligible for inclusion if they met criteria for dementia (defined as a score 1 or greater on the Clinical Dementia Rating scale^17^ and/or a score of 4 or greater on the Functional Assessment Staging of Alzheimer's Disease [FAST] scale).^18^

### 2.3 Descriptive data

Demographic information was recorded regarding sample characteristics, including age, gender, ethnicity, and severity of dementia.

**RESEARCH IN CONTEXT**

1. **Systematic review**: The authors reviewed the literature using traditional (PubMed, Medline, EMBASE) sources and meeting abstracts and presentations. There is extensive literature regarding the impact of antipsychotic medication and neuropsychiatric symptoms in dementia, and several programs showing benefit of psychosocial interventions, but none that evaluate a remote‐delivery model for nursing home training in a pandemic context.
2. **Interpretation**: This review highlighted the clear need to translate current evidence‐based training programs for digital and remote delivery, and led to a hypothesis that a remote delivery model would confer benefits to people with dementia in nursing homes.
3. **Future directions**: The study clearly demonstrates the benefits of the remote‐ and digital‐delivery model of the iWHELD for people with dementia living in nursing homes and highlights the opportunity for large‐scale rollout of evidence‐based programs to improve quality of life, even in a pandemic situation.

## 3 INTERVENTION

The iWHELD intervention is an adapted version of the previously evaluated WHELD program, aimed to support nursing home staff in PCC practices and promote personalized activities and social interactions, providing alternatives to psychotropic medications. The intervention included a 1‐month orientation phase to assess learner needs and introduce the program, followed by a 4‐month off‐site training phase with 1 day per month of PCC classroom‐based sessions led by a WHELD trainer, incorporating didactic learning, experiential activities, and implementation planning. The remaining 4 months featured regular on‐site consultation sessions with each care home, providing continuous support for PCC implementation. For detailed information about the WHELD program, please refer to the National Institute of Health Research report.^19^

In this study the adapted iWHELD program was delivered utilizing a digital platform with live virtual coaching sessions led by trained iWHELD Coaches; the program aimed to support nursing homes during the COVID‐19 pandemic and improve the health and well‐being of residents. The goal of iWHELD was to enhance nursing home practices with evidence‐based tools and materials, rather than increase workload while fostering collaborative discussion and sharing of best practices among peers.

Prior to baseline data collection, nursing home managers identified up to four staff members as iWHELD Champions, representing various caregiving roles, including managers, nursing staff, care assistants, and activity coordinators, to facilitate the implementation of PCC practices.

During the 16‐week intervention period, iWHELD Champions received dedicated guidance and support from an iWHELD Coach, utilizing evidence‐based tools and resources available on the iWHELD digital hub. Nursing homes were randomly grouped into coaching clusters, with up to five homes in each group, to encourage collaboration and knowledge exchange. Weekly, 45‐minute interactive coaching sessions conducted virtually provided Champions an opportunity to share experiences and best practices. Offline takeaway tasks were carried out to reinforce the training.

The iWHELD digital hub served as a central platform, providing prompts for reflective practice, continuous support, and networking opportunities through a peer learning platform, all overseen by the iWHELD coaching team. To ensure program consistency, coaches received individual and peer supervision from experienced therapists within the senior research team. Additionally, coaches underwent comprehensive training that encompassed evidence‐based PCC practices, coaching approaches, and the effective orientation to the newly launched iWHELD digital hub. The delivery of the intervention in each nursing home was the responsibility of the iWHELD Champions. Key components of the iWHELD program are summarized in Box 1. The goal was to enhance practice and care planning, not to add specific additional work elements. The control group received TAU.

**Components of the iWHELD program**

iWHELD Intervention Details 

1. Orientation Phase
2. Duration: 1 to 2 weeks leading up to the intervention.
3. Delivered by: iWHELD Coaches.
4. Participants: Nursing home teams, including managers and nominated staff champions.
5. Aim: Conduct 1:1 or group sessions to introduce the iWHELD program, provide onboarding to the digital platform, and explain access procedures using telephone and subsequent Zoom calls. Participants received onboarding guides via email.
6. Intervention Delivery Phase
7. Duration: 16 weeks.
8. Delivered by: Trained iWHELD Coaches.
9. Participants: Up to four staff members selected as iWHELD Champions in each nursing home.
10. Aims: Weekly coaching sessions with peer nursing homes covering key topics such as understanding person‐centered care, developing strengths‐based care plans and tailored social activities, understanding unmet needs, and evidence‐based practices for antipsychotic medication use.
11. Delivery Style and Format
12. Coaching Model: Empowering learners through interactive discussions, experiential activities, and goal setting during supportive coaching sessions conducted virtually via Zoom promoting a learner‐centric approach
13. Weekly 45‐Minute Coaching Sessions: Participants engage in live, virtual coaching with up to 5 peer nursing homes per session, fostering collaboration and sharing best practices.
14. Take‐Away Tasks: Participants are encouraged to carry out tasks in their nursing homes to apply the learning into practice.
15. Reflective Practice: Prompts for reflective practice, ongoing support, and networking are available on the digital hub, supported by the iWHELD coaching team.
16. Personalized: The combination of the coaching model, guided program, and accessible digital hub leads to a tailored and impactful intervention, catering to the unique needs of each nursing home and champion.
17. Supervision: iWHELD Coaches receive individual and peer supervision from experienced therapists within the senior research team to ensure consistent program delivery.
18. Digital Hub Accessibility
19. The iWHELD digital hub offers 24/7 accessibility, enabling Champions to access all program materials conveniently and at their own pace, regardless of time or location.
20. Unrestricted Access: Participants can freely navigate and utilize the available materials without any content gating based on completion status, fostering a flexible and self‐directed learning experience. The hub provides relevant evidence‐based tools, materials, and resources to enhance knowledge and skills in person‐centered care.

### 3.1 Outcome measures

All outcome measures were assessed prior to randomization and post‐intervention by a trained research assistant, who conducted interviews virtually or by telephone with a member of staff with regular contact with individual participants. The primary outcome was QoL, measured by the overall QoL item on the DEMQOL‐Proxy.^20^ The secondary outcome measures included the EuroQol‐5 Dimension (EQ‐5D‐5L) as an additional measure of QoL. Other secondary outcomes included agitation evaluated with domain C of the Neuropsychiatric Inventory–Nursing Home Version (NPI‐C), with a threshold of >3 to indicate clinically significant agitation,^21^ and the use of psychotropic medication which included atypical antipsychotics, hypnotics, antidepressants, and anxiolytics. Prescriptions within 4 weeks of baseline and follow‐up were recorded from nursing home medication administration charts.

### 3.2 Consent, randomization, and blinding

Consent was obtained prior to baseline. Participants with mental capacity provided consent for their own participation. Consent was provided by next of kin or an appropriate legal representative when individuals did not have mental capacity to consent for themselves. The University of Exeter Clinical Trials Unit, who were independent of the trial team, randomized nursing homes to intervention or control groups immediately after baseline data collection with RedCAP Cloud software. This system is validated to ISO27001 standards, backed up and maintained in Europe. A minimization algorithm was used to balance the treatment arms for key parameters: size of the nursing home (<30 or ≥30 residents), participation in iWHELD digital hub beta testing, and whether participating nursing homes had experienced a COVID‐19 outbreak.

Researchers completing follow‐up assessments were blind to treatment allocation. Every attempt was made to control accidental un‐blinding by minimizing contact between iWHELD Coaches and the researchers collecting outcome data and with clear instructions to researchers and nursing home staff to not discuss treatment allocation.

### 3.3 Sample size

As a cluster trial, power was maximized by having a larger number of clusters with a smaller number of participants in each cluster. Assuming a Cohen's d of 0.25 and an interclass correlation of 0.05, consistent with our previous clinical trials,^22^ and an average of five residents sampled per nursing home (coefficient of variation of cluster size = 0.3), 63 nursing homes per arm were required to provide 80% power at a 5% significance level. Based upon an assumed dropout rate of no more than 15% of nursing homes, a recruitment target of 75 nursing homes per arm was stipulated, with a total target of 150 homes. All residents from a single nursing home are counted as a cluster.

### 3.4 Data analysis

**FIGURE 1:**
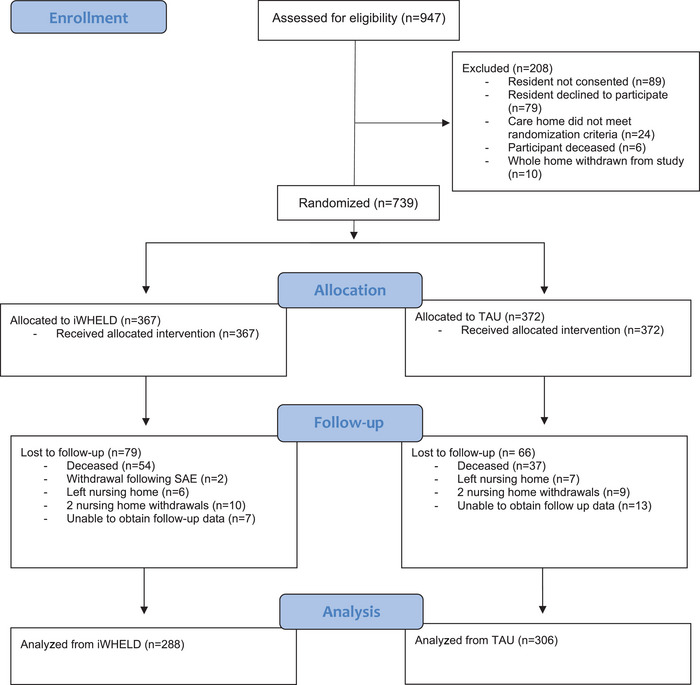
CONSORT flow diagram showing flow of participants through the trial. SAE, serious adverse event; TAU, treatment as usual.

Outcome measures for the study were assessed at baseline and at 16 weeks. The Consolidated Standards of Reporting Trials (CONSORT) diagram is presented in Figure 1. Baseline characteristics were summarized with descriptive statistics to determine whether there were imbalances between treatment groups at baseline. Continuous variables were summarized with the mean and standard deviation (SD) and categorical data were summarized as a number and percentage. All outcome measures were analyzed using an intention to treat approach. The primary outcome was calculated as the difference in change from baseline to 16 weeks between the treatment groups on QoL measured by the DEMQOL‐Proxy summary item for overall QoL.^23^ Statistical evaluation of the primary outcome used one‐way analysis of variance (ANOVA), adjusted for baseline levels of the outcome measure and age. The same approach was utilized for the evaluation of the secondary measure of QoL, the EQ‐5D‐5L. Differences in the number of participants at follow‐up with clinically significant agitation and differences in taking psychotropic drugs between treatment arms were compared at baseline and follow‐up using the Fisher's exact test. An additional secondary question analyzed whether the intervention conferred benefit in people who experienced COVID‐19 during the 16‐week period of the trial. This was evaluated using the same ANOVA approach. Further exploratory evaluations of QoL in at‐risk groups, including individuals taking psychotropic drugs, those with clinically significant agitation (NPI‐C > 3), and those suspected or confirmed to have had COVID‐19 during the 16 weeks of the trial were undertaken using the same ANOVA model. All statistical analyses were undertaken using SPSS Version 28. DEMQOL‐Proxy scores were converted to a scale of 0 to 100, with higher scores corresponding to a higher QoL to enable direct comparability between DEMQOL‐Proxy and EQ‐5D‐5L scores.

## 4 RESULTS

### 4.1 Cohort characteristics

A total of 739 participants were assessed at baseline, with a mean age of 85.5 years (SD 8.0). Sixty‐nine percent were female and 72% had moderately severe or severe dementia according to ratings on the FAST scale. The majority were Caucasian. Two hundred and ninety‐three (41%) participants were taking one of four pre‐specified psychotropic medications (antipsychotics, anxiolytics, hypnotics, or antidepressants), 216 (29%) participants had clinically significant agitation, and 307 (42%) participants had “fair” or “poor” QoL at baseline using the DEMQOL‐Proxy measure of overall QoL. There was a significantly higher rate of agitation at baseline in the intervention arm (33% in the intervention arm vs 27% in the TAU arm) and there was a non‐significant numerical imbalance in ethnicity with a more diverse resident population in the intervention arm. All other characteristics were balanced between treatment groups. The baseline characteristics and comparison between the treatment group and the TAU group are shown in Table 1.

**TABLE 1:** Cohort characteristics at baseline showing breakdown of age, gender, and ethnicity.

Patient characteristics	COVID iWHELD *n =* 367	TAU *n =* 372	Group comparison
Age (Mean [SD])	85.8 (7.55)	85.3 (8.45)	t = 0.97, *p =* 0.33
Gender			
Male	106 (28.9%)	120 (32.3%)	*χ* ^2^ = 1.96, df = 1, *p =* 0.38
Female	260 (70.8%)	252 (67.7%)	
Prefer not to say	1 (0.3%)	0 (%)	
Ethnicity			
Black	7 (1.9%)	11 (2.9%)	*χ* ^2^ = 10.39, df = 1, *p =* 0.07
Asian	0	6 (1.6%)	
White	357 (97.3%)	350 (94.1%)	
Mixed/Other	3 (0.8%)	5 (1.4%)	
Quality of life (DEMQOL‐Proxy summary score [%])	38 (10.3%)	33 (9%)	*χ* ^2^ = 3.1, df = 3, *p =* 0.37
Very Good	171 (46.3%)	187 (51%)	
Good	118 (32.0%)	99 (27%)	
Fair Poor	42 (11.4%)	48 (13.1%)	
Clinically‐significant agitation (NPI‐C > 3)	95 (26.8%)	121 (33.2%)	*χ* ^2^ = 4.3, df = 1, *p =* 0.04
Psychotropic medications (antipsychotics, anxiolytics, hypnotics, antidepressants combined)	155 (42.6%)	138 (39.0%)	*χ* ^2^ = 1.99, df = 1, *p =* 0.37

*Note*: Missing data are as follows: DEMQOL‐Proxy *n =* 3, age *n =* 1, gender *n =* 0, ethnicity *n =* 0, agitation *n =* 5, psychotropic medications *n =* 22.

Abbreviations: NPI‐C, Neuropsychiatric Inventory, domain C; TAU, treatment as usual.

A total of 594 (80.3%) participants completed follow‐up (iWHELD *n* = 288, TAU *n* = 306). The majority of non‐completions were attributed to participant mortality, which is expected in the case of a frail group of older nursing home residents with dementia. Detailed information regarding participant flow is shown in the CONSORT chart presented in Figure 1.

### 4.2 Impact of the iWHELD intervention on QoL in nursing home residents

In the primary analysis, residents in nursing homes receiving the iWHELD intervention had a significant benefit in QoL on the DEMQOL‐Proxy compared to residents in the TAU group over the 16‐week intervention period (F = 4.3, *p* = 0.04). This benefit in QoL was supported by a significant advantage in the change in QoL for the intervention group compared to the TAU group on the EQ‐5D‐5L as a second independent measure of QoL (F = 6.5, *p* = 0.01). The results are shown fully in Table 2.

**TABLE 2:** Main analysis outcomes from one‐way ANOVA (DEMQOL‐Proxy, EQ‐5D, agitation) and chi‐square (psychotropic drug use) comparing the iWHELD intervention with treatment as usual.

	Intervention group (*n*)	Control group (*n*)	F	χ^2^	*p*	Mean difference (SE)	95% CI for mean difference	Effect size
**ANOVA**								
DEMQOL‐Proxy (overall QoL)	288	306	4.3	–	0.04	4.99 (2.40)	0.28 to 9.70	0.15
EQ‐5D	255	290	6.45	–	0.01	4.68 (1.84)	1.06 to 8.30	0.21
**Fisher's exact test**								
Clinically significant agitation at follow‐up	59 (22.6%)	79 (37%)			FET *p =* 0.13			
Psychotropic drug use	Meds: 86 No meds: 165 (34%)	Meds: 126 No meds: 169 (43%)	–		FET *p =* 0.04			

Abbreviations: ANOVA, analysis of variance; CI, confidence interval; EQ‐5D, EuroQol‐5 Dimension; FET, Fisher's exact test; QoL, quality of life.

### 4.3 Impact of the iWHELD intervention on secondary outcomes

There was no significant difference in change in agitation or clinically significant agitation across the study between the iWHELD intervention and TAU groups, although the prevalence of clinically significant agitation was almost 15% lower in the iWHELD group in comparison to those allocated to TAU (37.1% vs 22.6%). There was no difference in the use of psychotropic drugs between treatment groups at baseline (*χ* ^2^ = 1.99, *p* = 0.37), but there was a significantly lower use of psychotropics in the iWHELD intervention group compared to the TAU group at follow‐up (*χ* ^2^ = 4.08, *p* = 0.044). The main results are described in more detail in Table 2. A further binary logistic regression looking at individual psychotropic drugs at follow‐up confirmed a significant overall reduction in psychotropic drugs (*p* = 0.015), but none of the individual drugs differed significantly between the treatment arms.

### 4.4 Further analysis: Participants with suspected or confirmed COVID‐19 during the RCT

Over the 16 weeks of the trial, 200 participants developed COVID‐19, comprising 30% (*n* = 86) in the iWHELD group and 38% (*n* = 114) in the TAU group (*χ* ^2^ = 3.5, df = 1, *p* = 0.06) among participants completing the follow‐up. At baseline the participants who subsequently contracted COVID‐19 in each treatment arm had similar characteristics with respect to age (iWHELD mean 85.2, SD 8.5 vs TAU mean 85.6, SD 8.1, t = 0.35, *p* = 0.73) and gender (iWHELD *n* = 68 [79%] vs TAU *n* = 81 [71%], *χ* ^2^ = 1.68, *p* = 0.20). Levels of psychotropic medication use (iWHELD *n* = 31 [37.8%] vs TAU *n* = 54 [47.4%], *χ* ^2^ = 1.78, *p* = 0.18) and clinically significant agitation (iWHELD *n* = 22 [25.6%] vs TAU *n* = 35 [30.7%], *χ* ^2^ = 0.64, *p* = 0.43) did not differ significantly between groups.

In comparison to the TAU group, participants who developed COVID‐19 during the period of the trial assigned to the iWHELD intervention showed a significant 4.81 (SE 1.30) point benefit in QoL as measured by the EQ‐5D‐5L (Cohen's d effect size 0.27) and a 12% lower frequency of clinically significant agitation at follow‐up (Fisher's exact test, *p* = 0.065). There was also a numerical advantage on the DEMQOL‐Proxy, as another measure of QoL, which did not achieve statistical significance. There was no significant difference in the use of psychotropic medication in this subgroup between treatment groups (see Table 3).

**TABLE 3:** Outcomes in participants with reported or confirmed COVID 19 during the trial.

Measure	*N*	Mean difference (SE) (IWHELD v TAU)	95% CI for mean difference	*p*	Cohen's d
**ANOVA**					
DEMQOL‐Proxy (overall QoL)	iWHELD = 86 TAU = 114	5.42 (4.08)	−2.62 to 13.47	0.19	0.16
EQ‐5D	iWHELD = 78 TAU = 109	9.06 (3.33)	2.49 to 15.62	0.01	0.41
**Chi‐square**					
Clinically significant agitation at follow‐up (NPI‐C > 3) total change	iWHELD = 81 TAU = 110	15 (18.5%)	34 (30.9%)	FET 0.065	
Combined psychotropic medications at follow‐up	iWHELD = 77 TAU = 110	25 (32.4%)	46 (41.8%)	FET 0.13	

Abbreviations: ANOVA, analysis of variance; CI, confidence interval; EQ‐5D, EuroQol‐5 Dimension; FET, Fisher's exact test; NPI‐C, Neuropsychiatric Inventory, domain C; QoL, quality of life; TAU, treatment as usual.

### 4.5 Exploratory analyses

Further exploratory analyses were undertaken to evaluate the impact of iWHELD in subgroups at risk of lower QoL. These subgroups were defined as residents with clinically significant agitation (NPI‐C > 3) or those receiving a psychotropic drug at baseline. Significant benefits, more substantial than seen in the overall trial cohort, were seen in QoL for residents in both at‐risk subgroups in the intervention arm compared to those in the TAU group (see Table 4).

**TABLE 4:** Change in QoL on the DEMQOL‐Proxy and EQ‐5D measures in residents at risk of worsening of neuropsychiatric symptoms.

Sub‐group	F	*p*‐value	Mean difference (SE)	95% CI for mean difference	Cohen's d
**Clinically significant agitation (NPI‐C > 3)**					
DEMQOL‐Proxy (overall QoL) (*n =* 207)	4.07	0.06	7.84 (4.19)	−0.42 to 16.09	0.23
EQ‐5D (*n =* 182)	6.5	0.01	8.67 (3.49)	1.79 to 15.56	0.39
**Taking psychotropics**					
DEMQOL‐Proxy (overall QoL) (*n =* 243)	4.1	0.01	9.38 (3.71)	2.08 to 16.68	0.28
EQ‐5D (*n =* 216)	6.5	0.02	6.97 (3.05)	0.96 to 12.97	0.31

Abbreviations: CI, confidence interval; EQ‐5D, EuroQol‐5 Dimension; FET, Fisher's exact test; NPI‐C, Neuropsychiatric Inventory, domain C; QoL, quality of life

## 5 DISCUSSION

The iWHELD RCT was successfully delivered to 739 nursing home residents with dementia across 149 UK nursing homes. The study demonstrated significant benefits on two independent measures of QoL amongst residents living in nursing homes receiving the iWHELD intervention over a 16‐week period. Nursing homes receiving the iWHELD program also achieved a significant 20% reduction in use of psychotropic medications compared to the TAU group, with no detriment to agitation across the period of the trial. The QoL benefits of iWHELD in comparison to TAU were greater in people with existing clinically significant agitation and those taking psychotropic medications at baseline. These groups represent the individuals with the highest level of need.

Although there was no significant overall improvement in agitation for people receiving iWHELD to those allocated to TAU, the prevalence of clinically significant agitation at follow‐up was almost 15% lower in the iWHELD group, and there was a clinically meaningful benefit in QoL in participants with agitation and residents taking psychotropic medication in the intervention group. Improving well‐being and QoL is a key goal of any therapy for people with dementia. However, despite some modest improvements in symptoms, treatment with atypical antipsychotics often has a detrimental impact on QoL in these individuals.^15^ The current findings suggest that as new and more effective pharmacological treatments for neuropsychiatric symptoms emerge, a combined approach with effective non‐pharmacological approaches may have the best impact in maintaining or improving QoL.

Although psychotropic drug use was significantly reduced in nursing homes receiving iWHELD compared to TAU, there was no specific reduction in use of any individual class of psychotropic medication including atypical antipsychotics. This is contrary to the previous in‐person delivery of the WHELD program which resulted in a more substantial 50% reduction in antipsychotic medications.^22^ This difference may be due to the longer duration of the earlier study (9 months compared to 4 months) and the concurrent delivery of a primary care training program in the previous pre‐pandemic trial. Potential augmentation of iWHELD with training for primary care physicians and other primary care practitioners will be an important consideration going forward.

Uniquely, this study included an additional analysis focusing specifically on people who experienced COVID‐19 during the trial. The results indicated a significant impact on QoL as measured by the EQ‐5D‐5L scale, with the iWHELD intervention turning a decline in QoL into an improvement, conferring a nine‐point benefit for individuals contracting COVID‐19 compared to those receiving TAU, with a Cohen's d effect size of 0.41. Interestingly, there was a near‐significant reduction in people contracting COVID‐19 in the active intervention group which may warrant further investigation as to the potential wider health benefits of the iWHELD program, particularly with regard to COVID‐19. Whilst these findings are encouraging, additional work is needed to explore these possible associations further.

Both remote and in‐person iterations of the WHELD intervention have demonstrated effectiveness in improving QoL for individuals with dementia and reducing psychotropic medication use, providing a choice of effective approaches to nursing homes depending on which option best matches their care practices and the available resources. When considering scalability, however, the fully remote, digital format of iWHELD offers enhanced accessibility, enabling participation without geographical limitations. Its 24/7 access to materials supports night shift staff and fosters personalized learning, and weekly coaching sessions with peer nursing homes promote collaboration and knowledge exchange, making iWHELD a more versatile and cost‐efficient approach to enhance the well‐being of individuals with dementia in nursing homes.

This study provides data from a large‐scale trial which recruited, engaged, and retained nursing homes during the COVID‐19 pandemic at a time when most other research in the sector was halted.^24^ Eighty percent of participants completed the trial, which compares favorably to other studies in frail nursing home populations. The data outputs were collected and delivered in adherence to the planned protocol and the study met its anticipated power despite the unprecedented challenges posed by the environment at the time. As the study was conducted during the pandemic at a time of great stress for nursing homes, assessments were conducted virtually, and the number of secondary outcomes was restricted to reduce burden. There were more missing data for psychotropic drugs (3% missing) than would be expected in non‐virtual studies. Of note, there was a modest but significant increase in participants with clinically significant agitation at baseline in the intervention group compared to TAU. Importantly, agitation at baseline was used as a covariate in each analysis focusing on agitation as an outcome. Overall, the study has provided significant, meaningful results with relevance beyond the period of the pandemic.

The findings from this trial demonstrate the substantial benefits of iWHELD as an innovative, practical, accessible, and scalable solution to enhance the QoL for residents in nursing home settings. iWHELD conferred significant overall benefits in improving QoL and reducing psychotropic drug use. Notably, the program yielded the most substantial benefits for residents who contracted COVID‐19 during the study, those with agitation at baseline, and participants already prescribed psychotropic medications. These findings emphasize the potential of iWHELD to address critical needs in diverse nursing home settings, positioning it as a valuable intervention to enhance the well‐being and care outcomes of residents of participating homes.

## CONFLICTS OF INTEREST STATEMENT

CB has received consulting fees from Acadia pharmaceutical company, AARP, Addex pharmaceutical company, Eli Lily, Enterin pharmaceutical company, GW Pharmaceuticals, H. Lundbeck pharmaceutical company, Novartis pharmaceutical company, Janssen Pharmaceuticals, Johnson and Johnson pharmaceuticals, Novo Nordisk pharmaceutical company, Orion Corp pharmaceutical company, Otsuka America Pharm Inc, Sunovion Pharm. Inc, Suven pharmaceutical company, Roche pharmaceutical company, Biogen pharmaceutical company, Synexus clinical research organization and TauRX pharmaceutical company and research funding from Synexus clinical research organization, Roche pharmaceutical company, Novo Nordisk pharmaceutical company and Novartis pharmaceutical company. AC discloses financial relationships with Suven and Janssen pharmaceutical companies for consultancy work; MSM has received consulting fees from Acadia pharmaceutical company, Avanir company, Medesis Pharma, Otsuka company, Roche pharmaceutical company, Biogen pharmaceutical company and Biogen pharmaceutical company. JC has provided consultation to Acadia, Actinogen, Acumen, Alpha Cognition, Aprinoia, AriBio, Artery, Biogen, BioVie, Cassava, Cerecin, Diadem, EIP Pharma, Eisai, GemVax, Genentech, GAP Innovations, Janssen, Jocasta, Karuna, Lilly, Lundbeck, LSP, Merck, NervGen, Novo Nordisk, Oligomerix, OptoCeutics, Ono, Otsuka, PRODEO, Prothena, ReMYND, Roche, Sage Therapeutics, Signant Health, Simcere, Sunbird Bio, Suven, SynapseBio, TrueBinding, Vaxxinity, and Wren pharmaceutical, assessment, and investment companies. JC owns the copyright of the Neuropsychiatric Inventory. JM, WH, GW, DA, CF, JF, LC, BWC, EMC, MSM, AS, and KM report no financial relationships with commercial interests. Author disclosures are available in the supporting information.

## CONSENT STATEMENT

We confirm that all human subjects provided informed consent for this study. In cases where capacity was lacking consent was provided in consultation with a designated consultee.

## Supporting information

Supporting Information
